# SLC25A1 promotes tumor growth and survival by reprogramming energy metabolism in colorectal cancer

**DOI:** 10.1038/s41419-021-04411-2

**Published:** 2021-11-27

**Authors:** Ying Yang, Jiaxing He, Bo Zhang, Zhansheng Zhang, Guozhan Jia, Shiqi Liu, Tao Wu, Xianli He, Nan Wang

**Affiliations:** Department of General Surgery, Tangdu Hospital, the Air Force Medical University, Xi’an, 710038 China

**Keywords:** Colon cancer, Colon cancer

## Abstract

Abnormal lipid metabolism has been commonly observed in various human cancers, including colorectal cancer (CRC). The mitochondrial citrate carrier SLC25A1 (also known as mitochondrial citrate/isocitrate carrier, CIC), has been shown to play an important role in lipid metabolism regulation. Our bioinformatics analysis indicated that SLC25A1 was markedly upregulated in CRC. However, the role of SLC25A1 in the pathogenesis and aberrant lipid metabolism in CRC remain unexplored. Here, we found that SLC25A1 expression was significantly increased in tumor samples of CRC as compared with paired normal samples, which is associated with poor survival in patients with CRC. Knockdown of SLC25A1 significantly inhibited the growth of CRC cells by suppressing the progression of the G1/S cell cycle and inducing cell apoptosis both in vitro and in vivo, whereas SLC25A1 overexpression suppressed the malignant phenotype. Additionally, we demonstrated that SLC25A1 reprogrammed energy metabolism to promote CRC progression through two mechanisms. Under normal conditions, SLC25A1 increased de novo lipid synthesis to promote CRC growth. During metabolic stress, SLC25A1 increased oxidative phosphorylation (OXPHOS) to protect protects CRC cells from energy stress-induced cell apoptosis. Collectively, SLC25A1 plays a pivotal role in the promotion of CRC growth and survival by reprogramming energy metabolism. It could be exploited as a novel diagnostic marker and therapeutic target in CRC.

## Introduction

Lipid metabolism reprogramming has been recognized as a hallmark of malignancy, which provides rapidly proliferating cancer cells with fatty acids for membrane biogenesis, energy storage/production, and signal transduction [1–3]. Targeting dysregulated lipid metabolic pathways has been well recognized as a novel anti-cancer strategy, although not yet realized [4]. One of the key alterations in lipid metabolism that has been observed in many different cancer types is elevated de novo fatty acid synthesis [5]. The rate-limiting enzymes in fatty acid synthesis, such as fatty acid synthase (FASN) and acetyl-CoA carboxylase (ACC), have been reported to be overexpressed and involved in the promotion of cancer progression [6, 7]. Additionally, several previous studies also have reported increased fatty acid β-oxidation under metabolic stress in several types of cancer [8]. However, how cancer cells coordinate de novo fatty acid synthesis and fatty acid oxidation is still poorly understood.

Citrate provides the source of Ac-CoA for fatty acid synthesis in the cytoplasm, while in mitochondria, it enters the Krebs cycle for oxidative phosphorylation [9]. The solute family member *SLC25A1*, also known as mitochondrial citrate/isocitrate carrier (CIC), is the only known transporter of human citrate in the mitochondrial membrane. The functions of SLC25A1 consists the export of citrate from the mitochondria into the cytoplasm and the reverse import of cytosolic citrate into the mitochondria [10, 11]. Our bioinformatics analysis indicated that SLC25A1 was markedly upregulated in CRC. However, the roles of SLC25A1 in the pathogenesis and aberrant lipid metabolism in CRC have not been previously studied.

We wanted to question whether SLC25A1 overexpression contributes to the pathogenesis and aberrant lipid metabolism in colorectal cancer (CRC). In the present study, we explored the expression pattern and clinical relevance of SLC25A1 in CRC. In addition, the biological functions of SLC25A1 in the pathogenesis and aberrant lipid metabolism were also systematically investigated by knockdown and overexpression studies in CRC cells both in vitro and in vivo.

## Materials and methods

### Cell culture

Human CRC cell lines (SW480, HCT116, SW620, LOVO, LS174T and HT29) and one non-transformed colon epithelial cell line NCM460 were maintained in Dulbecco’s modified Eagle’s medium (Gibco, CA, USA) supplemented with 10% fetal bovine serum in a humidified atmosphere containing 5% CO_2_ at 37 °C. All cell lines were tested for absence of mycoplasma contamination and authenticated using the short tandem repeat (STR) method. For starvation treatment, CRC cells were cultured in glucose-free Dulbecco’s modified Eagle’s medium (Gibco, CA, USA) supplemented with 5% FBS.

### Gene silencing and overexpression

For knockdown of SLC25A1, small interfering RNA targeting the human SLC25A1 (sequence: 5’- CAGGGCCTGGAGGCGCACAdTT-3’) purchased from Ruibo Company. For the construction of shRNA expression vector targeting SLC25A1, a small hairpin RNA (shRNA) containing specific sequences targeting the human SLC25A1 (sequence: 5’- CAGGGCCTGGAGGCGCACAdTT-3’) was cloned into the pSilencer 3.1-H1 puro (Thermo Fisher Scientific). For overexpression of SLC25A1, the open reading frame (ORF) of human SLC25A1 was cloned into a pcDNA3.1 vector, which was followed by transfection with Lipofectamine 2000 (Invitrogen).

For transfection, CRC cells were seeded in 6-well plates to about 70% confluence. Then, the plasmids or siRNA were respectively transfected into CRC cells using Lipofectamine 2000 Reagent (Invitrogen, 11668-027) according to the manufacturer’s instructions. Stable transfections were selected with G418 or puromycin (Thermo Fisher Scientific, 10131035 or A1113802) within 2 months.

### Quantitative real-time PCR

RNA in CRC cells was extracted using TROzol reagent (Invitrogen) and reversely transcribed into cDNA using a cDNA synthesis kit (Marligen Biosciences) according to the manufacturer’s instructions. SYBR Green PCR Master Mix (Takara) was used for quantitative real-time PCR (qRT-PCR). The forward primer sequence of *SLC25A1* is 5ʹ-CCCCATGGAGACCATCAAG-3ʹ. Reverse primer sequence of *SLC25A1* is 5ʹ-CCTGGTACGTCCCCTTCAG-3ʹ. The forward primer sequence of *ACTB* is 5ʹ-GGCTGTATTCCCCTCCATCG-3ʹ. The reverse primer sequence of *ACTB* is 5ʹ-CCAGTTGGTAACAATGCCATGT-3ʹ. The relative gene expression for each sample was normalized to that of *ACTB*.

### Western blot

Proteins extracted from CRC cells were separated by SDS-PAGE and transferred to PVDF membranes, followed by incubation with blocking buffer (5% nonfat dry milk in PBS), primary SLC25A1 (Proteintech, 15235-1-AP) and ACTB (Proteintech, 20536-1-AP) antibodies overnight at 4 °C. After washing with PBS buffer three times, the membranes were probed with a horseradish peroxidase-conjugated secondary antibody. The signaling was detected by chemiluminescence (Thermo Scientific). For isolation of mitochondria fraction from cytosolic fraction of CRC cells, a commercial Mitochondria/Cytosol Fractionation Kit (ab65320, abcam) was used following the manufacturer’s protocols.

### MTS and colony formation assays

One thousand CRC cells were seeded in 96-well plates (020096, Xinyou Biotech, Hangzhou, China). Cell viability was examined by incubation with MTS-PMS solution (Promega) by measuring the OD (490 nm) at the indicated time points following the manufacturer’s instructions.

### Apoptosis assay

For cell apoptosis analysis, an FITC apoptosis detection kit (BD Biosciences) was used. Cultured CRC cells were collected and suspended in binding buffer. After that, cells were incubated with annexin V-FITC and propidium iodide (PI) solutions at 28 °C for 10 min. The results were analyzed by flow cytometry.

### Immunohistochemical (IHC) staining analysis

A total of 298 (30 for qRT-PCR analysis, 268 for IHC analysis) primary CRC and paired adjacent non-tumor tissue samples were obtained from patients at the Second Affiliated Hospital of the Air Force Medical University in Xi’an, China. Written informed consent was obtained from all patients. The study was approved by the Ethics Committee of the Second Affiliated Hospital of the Air Force Medical University in Xi’an, China.

Formalin-fixed, paraffin-embedded CRC tissue sections were dewaxed and rehydrated, which was followed by antigen retrieval. The sections were then incubated with primary antibodies SLC25A1 (Proteintech, 15235-1-AP) and Ki-67 (Proteintech, 27309-1-AP) at 4 °C overnight. Signals were detected using an IHC detection kit (Invitrogen), followed by counter-staining with hematoxylin. The results were examined under a light microscope.

### Quantification of lipid content

Chloroform/methanol (2:1) was firstly used for lipid extraction from the homogenates of CRC cells. After that, commercially available EnzyChromTM free fatty acid (FFA), triglyceride (TG), phospholipids (PL) and cholesterol (Chol) detection kits (Bioassay Systems, USA) were used for evaluation of the contents of fatty acids, triglycerides, phospholipids, and cholesterol, according to the manufacturers’ protocols.

### BODIPY 493/503 staining for neutral lipid

For detection of neutral lipids, CRC cells were incubated with BODIPY 493/503 fluorescence dye (Invitrogen) for 2 h at 28 °C in the dark. The results were examined using an Olympus FV-1000 confocal microscope.

### MTS and colony formation assays

For the MTS assay, 1 × 10^3^ cells were seeded in 96-well plates (020096, Xinyou Biotech, Hangzhou, China) and cultured in a humidified atmosphere containing 5% CO_2_ at 37 °C. Cell viability was determined using a cell proliferation kit (Promega) every day for four days, following the manufacturer’s instructions.

For the colony formation assay, 1000 CRC cells were seeded in 6-well plates and cultured in a humidified atmosphere containing 5% CO_2_ at 37 °C for 15 days. Colonies were washed with PBS buffer, fixed with 4% paraformaldehyde, and stained with crystal violet.

### Cell migration and invasion assay

Cell migration and invasion abilities were determined using 24-well chambers coated with or without an extracellular matrix. CRC cells (2 × 10^4^ or 5 × 10^4^) were seeded into the upper chamber in serum-free DMEM. After 24 or 48 h of culture, the migrated and invaded cells were fixed in 4% paraformaldehyde and stained with crystal violet. The number of migrated and invaded cells was counted under a light microscope.

### Tumor xenograft mouse model

Male athymic BALB/c nude mice (five weeks old) were housed with a 12-h light/dark cycle at 25 ± 1 °C and were randomly divided into groups (six mice/group) before cell injection. Stable SLC25A1 knockdown or control CRC cells (5.0 × 10^6^ cells/mice) were subcutaneously injected into the dorsal right flank of the mice. Tumor size was determined weekly. The mice were euthanized four weeks post cell injection, and the tumors were collected. All animal experimental procedures were approved by the Institutional Animal Care and Use Committee of the Air Force Medical University in Xi’an, China.

### Fatty acid uptake assay

Fatty acid uptake was evaluated with a Free Fatty Acid Uptake Assay Kit (Abcam, ab176768) according to the manufacturer’s protocol. Briefly, CRC cells were plated in a 96-well plate at a density of 5 × 10^4^ cells/well and cultured for 10 h. Then, cells were preincubated in serum-free media for 1 h and subsequently incubated with fluorescent fatty acid. After 50 min, the fluorescence intensity was detected by a microplate fluorescence reader at 485/515 nm.

### Oxygen consumption rate (OCR) detection

A total of 1 × 10^4^ CRC cells were plated onto the XFp microplates. The cellular oxygen consumption rate (OCR) of CRC cells with different treatments was determined with an extracellular flux analyzer (Seahorse Bioscience) following the manufacturer’s protocols.

### Detection of mitochondrial respiratory chain complexes I–V activities

To detect the activities of five mitochondrial respiratory chain complexes of I–V, a commercial kit (Abcam, ab110419) was used according to the manufacturer’s protocol. The absorption values were measured with a Bio-Rad microplate reader for the relative activities of the five complexes of I–V.

### MitoTracker Green staining assay

Mitochondrial morphology was visualized under a confocal microscopy by staining with MitoTracker Green fluorescent dye (Molecular Probes, M7514) following the manufacturer’s instruction. Image J software was used to measure the length of mitochondria.

### Statistical analysis

All experiments were performed three times, each performed in triplicate. All values were expressed as the mean ± SEM. Differences between two or more variables were assessed by two-tailed Student’s *t*-test or one-way ANOVA with Tukey’s post-hoc test. Patient survival was calculated using the Kaplan–Meier method. SPSS software (17.0) was used for statistical analysis. *P* < 0.05 was defined as statistically significant.

## Results

### SLC25A1 expression is markedly elevated in CRC

**Fig. 1:**
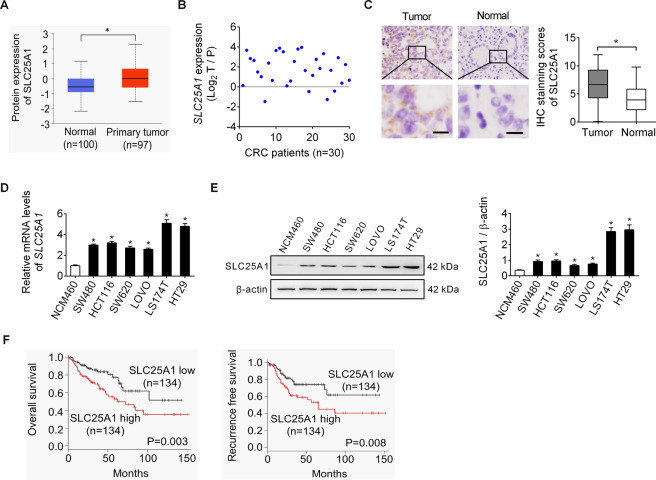
SLC25A1 expression is markedly elevated in CRC. **A** Bioinformatics analysis from UALCAN database was applied for protein expression of SLC25A1 in tumor and normal tissues of liver. **B** qRT-PCR analysis for mRNA expression of *SLC25A1* in tumor and matched adjacent nontumor tissues from 30 CRC patients. **C** Immunohistochemistry (IHC) staining of SLC25A1 in tumor and matched non-tumor tissues from another 268 CRC cohort. Scale bars, 10 μm. **D**, **E** qRT-PCR and western blot analysis for SLC25A1 expression in human CRC cell lines and non-transformed colon epithelial cell line. **F** Kaplan–Meier overall survival (left panel) and progression-free survival (right panel) analyses were applied according to SLC25A1 expression in CRC patients.

As shown in Fig. 1A, bioinformatics analysis based on the UALCAN database [12] revealed a significant upregulation of SLC25A1 in liver tumor tissues as compared to that in the matched normal tissues. Detection of SLC25A1 expression in tumor tissues and matched adjacent non-tumor tissues from 30 CRC patients by qRT-PCR analysis also indicated that the mRNA levels of *SLC25A1* were significantly higher in CRC samples than in adjacent non-tumor tissues (Fig. 1B). Consistently, immunohistochemistry (IHC) staining assay in another 268 CRC cohort also showed significantly higher SLC25A1 protein level in the tumor tissues than in the matched non-tumor tissues (Fig. 1C). In addition, qRT-PCR and western blot analysis also revealed a substantial increase of SLC25A1 expression in CRC cell lines as compared to non-transformed colon epithelial cell line (Fig. 1D, E). Correlation analysis between SLC25A1 expression and the clinicopathological features of CRC patients revealed that SLC25A1 expression was significantly positively correlated with tumor size (Supplementary Table 1). Kaplan–Meier survival analysis indicated that CRC patients with high SLC25A1 expression had significantly shorter survival time (Fig. 1F) than those with low SLC25A1 expression. Together, these findings highlight that SLC25A1 is upregulated in CRC and could serve as a survival predictor for patients with CRC.

### SLC25A1 promotes CRC cell growth in vitro and in vivo

**Fig. 2:**
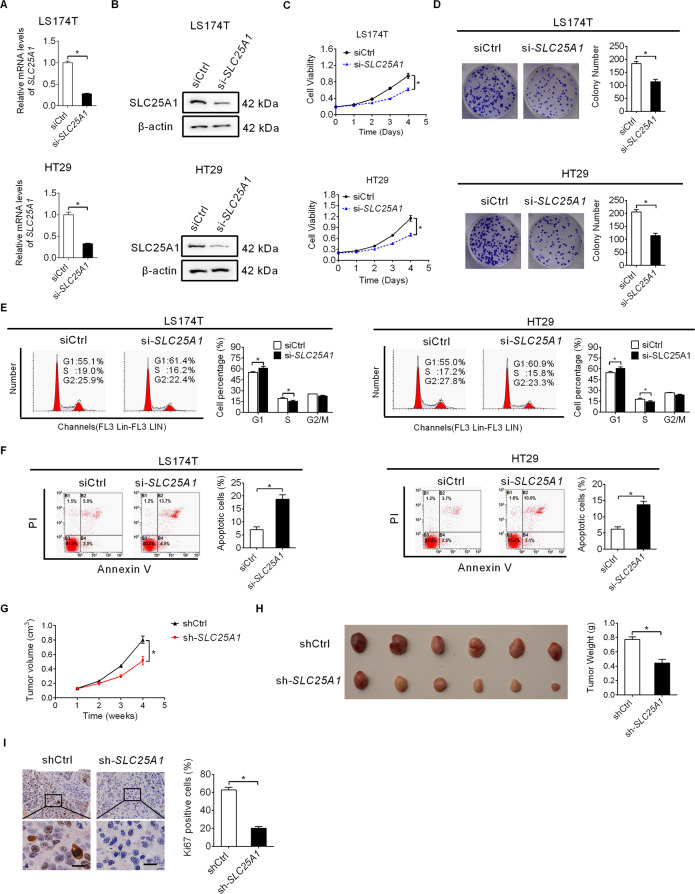
SLC25A1 promotes CRC cell growth in vitro and in vivo. **A**, **B** Knockdown of SLC25A1 was confirmed by qRT-PCR and western blot assays in LS174T and HT29 cells. **C** Cell proliferation was determined by MTS assay in LS174T and HT29 cells with SLC25A1 knockdown. **D** Colony formation assay in LS174T and HT29 cells with SLC25A1 knockdown. **E**, **F** Cell cycle and apoptosis assays in LS174T and HT29 cells with SLC25A1 knockdown. **G**, **H** The sizes and weights of xenograft tumors from nude mice (six mice per group) were compared between SLC25A1 knockdown and control groups. **I** IHC staining of ki-67 in xenograft tumors from the nude mice. Scale bars, 10 μm.

To investigate the role of SLC25A1 in CRC progression, chemical small interference RNA (siRNA) corresponding to SLC25A1 was synthesized to knockdown SLC25A1 expression in LS174T and HT29 cells with high endogenous SLC25A1 expression. qRT-PCR and western blot analysis confirmed that SLC25A1 was efficiently silenced in LS174T and HT29 cells (Fig. 2A, B). Our results showed that SLC25A1 knockdown markedly suppressed CRC cell proliferation and colony formation (Fig. 2C, D), while cell migration and invasion remained unchanged (Figure S1A, B). Considering that suppressed CRC growth may be caused by cell cycle arrest or/and increased apoptosis, we therefore determined the effect of SLC25A1 knockdown on cell cycle distribution and apoptosis using flow cytometry. Knockdown of SLC25A1 significantly induced G1 to S cell cycle arrest and apoptosis in LS174T and HT29 cells (Fig. 2E, F). We further evaluated the role of SLC25A1 in the promotion of CRC progression in vivo by injecting stable SLC25A1 knockdown LS174T cells (Figure S1C, D) into BALB/c mice. Stable knockdown of SLC25A1 by transfection with shRNA expression vector targeting SLC25A1 markedly decreased tumor growth rate and weight as compared to the control group (Fig. 2G, H). Additionally, tumor tissues developed from SLC25A1 knockdown LS174T cells had significantly lower expression of Ki67 than the control group (Fig. 2I). Moreover, consistent with the effects of transient transfection of siRNA targeting SLC25A1, stable knockdown of SLC25A1 by shRNA transfection also markedly decreased the proliferation and colony formation abilities of LS174T and HT29 cells (Figure S1E, F).

**Fig. 3:**
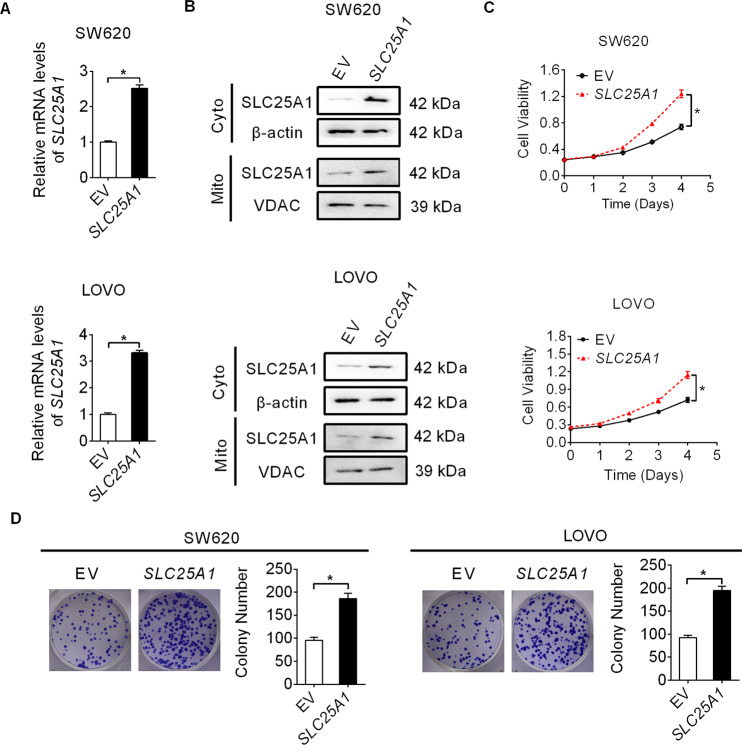
SLC25A1 overexpression promotes CRC cell growth. **A**, **B** Over-expression of SLC25A1 was confirmed by qRT-PCR and western blot analysis in SW620 and LOVO cells (Mito mitochondria, Cyto cytoplasm). **C** Cell proliferation was determined by MTS assay in SW620 and LOVO cells with SLC25A1 overexpression. **D** Colony formation assay in SW620 and LOVO cells with SLC25A1 overexpression.

Next, we overexpressed both cytoplasmic and mitochondrial fractions of SLC25A1 in SW620 and LOVO cells by transfection with the constructed SLC25A1 overexpression plasmid (Fig. 3A, B). As shown in Fig. 3C, D, SLC25A1 over-expression SW620 and LOVO cells exhibited a significant increase in cell proliferation and colony formation as compared to the control cells. Together, these data suggest SLC25A1 as an oncogene in CRC progression.

### SLC25A1 enhances fatty acid synthesis in CRC cells

**Fig. 4:**
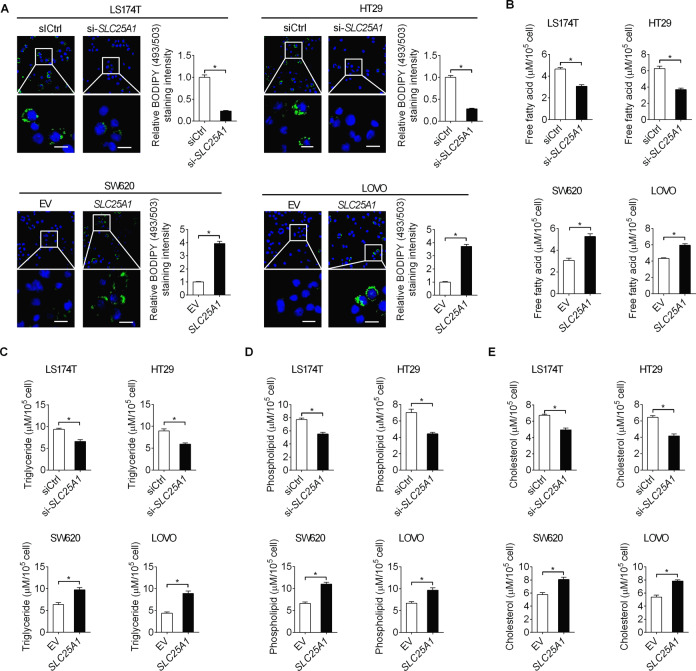
SLC25A1 enhances fatty acid synthesis in CRC cells. **A** Intracellular contents of neutral lipids were stained with the fluorescence lipophilic dye BODIPY 493/503 in CRC cells with different SLC25A1 expression levels. Scale bars, 10 μm. **B**–**E** Intracellular levels of free fatty acid (**B**), triglyceride (**C**), phospholipids (**D**) and cholesterol (**E**) were measured in CRC cells with different SLC25A1 expression levels.

Citrate provides the source of Ac-CoA for fatty acid synthesis. Since SLC25A1 is the only transporter of citrate in the mitochondrial membrane, we investigated whether SLC25A1 upregulation plays a role in the regulation of lipid biosynthesis in CRC cells. Detection of lipid content by the fluorescent lipophilic dye BODIPY 493/503 revealed that SLC25A1 knockdown markedly reduced the intracellular content of neutral lipids in LS174T and HT29 cells, whereas SLC25A1 overexpression elevated the intracellular content of neutral lipids in SW620 and LOVO cells (Fig. 4A). In addition, SLC25A1 knockdown also decreased intracellular levels of free fatty acids (Fig. 4B), triglycerides (Fig. 4C), phospholipids (Fig. 4D) and cholesterol levels (Fig. 4E) in LS174T and HT29 cells. By contrast, overexpression of SLC25 A1 elevated the levels of these lipids in SW620 and LOVO cells (Fig. 4B–E). Given that increased exogenous fatty acid (FA) uptake, another well characterized lipid metabolism alteration in human cancers, may also contribute to SLC25A1-induced accumulation of lipid content, we further evaluated the effect of SLC25A1 downregulation or upregulation on FA uptake in CRC cells. No significant change in FAs uptake was observed in SLC25A1 knockdown and overexpression cells, as compared with control cells (Figure S2), suggesting that SLC25A1 promotes lipid content accumulation in CRC cells mainly through increasing fatty acid synthesis.

### SLC25A1 promotes CRC growth by increasing fatty acid synthesis

**Fig. 5:**
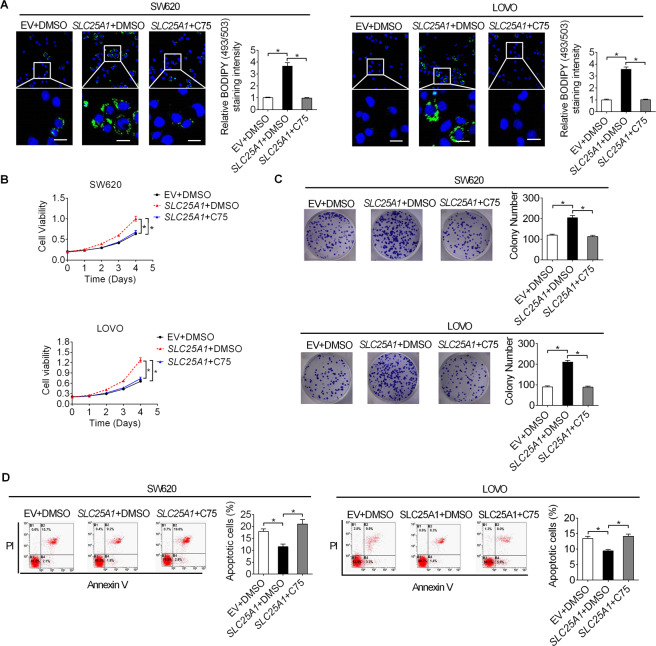
SLC25A1 promoted CRC growth by increasing fatty acid synthesis. **A** Intracellular contents of neutral lipids were stained with the fluorescence lipophilic dye BODIPY 493/503 in CRC cells treated with C75. Scale bars, 10 μm. **B** Cell proliferation was determined by MTS assay in CRC cells treated with C75. **C** Colony formation assay in CRC cells treated with C75. **D** Flow cytometry analysis for cell anostosis in CRC cells treated with C75.

Considering that de novo fatty acid synthesis plays an important role in meeting the biosynthetic demands for tumor cell growth, we hypothesized that upregulation of SLC25A1 might promote tumor growth of CRC by increasing fatty acid synthesis. To test this hypothesis, fatty acid synthesis was suppressed by the inhibition of the key enzyme fatty acid synthase (FASN) by treatment with C75 (an FASN inhibitor). Detection of lipid content confirmed that treatment with C75 significantly decreased the intracellular lipid content in LS174T cells with SLC25A1 overexpression (Fig. 5A), indicating the successful suppression of fatty acid synthesis in CRC cells by C75 treatment. As shown in Fig. 5B, C, forced expression of SLC25A1 significantly promoted proliferation and colon formation, which were markedly attenuated by treatment with C75. As expected, C75 treatment also restored SLC25A1 overexpression-suppressed apoptosis of CRC cells (Fig. 5D).

### SLC25A1 increased oxidative phosphorylation (OXPHOS) to promote cell survival during metabolic stress

**Fig. 6:**
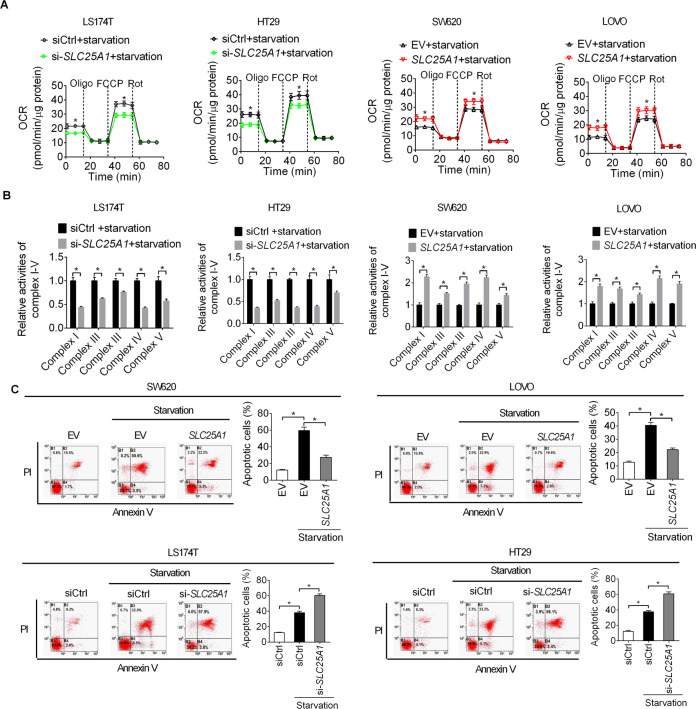
SLC25A1 increased oxidative phosphorylation (OXPHOS) to promote cell survival during metabolic stress. **A** Oxygen consumption rate (OCR) was determined in CRC cells during metabolic stress (glucose starvation). **B** Activities of respiratory chain complexes I–V were determined in CRC cells during metabolic stress (glucose starvation). **C** Flow cytometry analysis for the role of SLC25A1 in the regulation of CRC cell apoptosis during metabolic stress (glucose starvation).

The functions of SLC25A1 consists the export of citrate from the mitochondria into the cytoplasm for fatty acid synthesis and reverse import of cytosolic citrate into the mitochondria for OXPHOS [13]. Therefore, we next assessed the role of SLC25A1 in the oxidative phosphorylation of CRC cells. No significant changes in the oxygen consumption rate (OCR) and activities of respiratory chain complexes I–V were observed when SLC25A1 was knocked-down or overexpressed (Figure S3A, B) at normal conditions. Nevertheless, under energy stress (glucose starvation treatment), SLC25A1 knockdown markedly decreased OCR and activities of respiratory chain complexes I–V in LS174T and HT29 cells, while SLC25A1 overexpression significantly elevated OCR and activities of respiratory chain complexes I–V in SW620 cells (Fig. 6A, B). These results suggest that SLC25A1 may promote CRC cell survival under energy stress conditions. To test this hypothesis, cell apoptosis was assessed by flow cytometry in LS174T under energy stress. As shown in Fig. 6C, CRC cells transfected with empty vector (EV) under energy stress (absence of glucose in cell culture medium) showed a significant increase in cell apoptosis compared with CRC cells transfected with empty vector (EV) in the presence of glucose, while overexpression of SLC25A1 significantly attenuated energy stress-induced cell apoptosis. By contrast, knockdown of SLC25A1 remarkably aggravated energy stress-induced cell apoptosis. Together, these data suggest that SLC25A1 protects CRC cells from energy stress-induced cell apoptosis by increasing oxidative phosphorylation (OXPHOS).

Mitochondrial are highly dynamic organelles involved in energy metabolism. We therefore explored the potential involvement of mitochondrial morphological change in SLC25A1-regulated energy metabolism in CRC cells. We did not observe any significant change in mitochrodria morphology following SLC25A1 knockdown or overexpression both under normal or energy stress conditions (Figure S3C), suggesting that SLC25A1 reprogrammed energy metabolism in CRC cells may not through modulation of mitochondrial morphology.

## Discussion

The solute family member SLC25A1, also known as mitochondrial citrate/isocitrate carrier (CIC), is the only known transporter of human citrate in the mitochondrial membrane [13]. Here, we found that SLC25A1 expression was remarkably upregulated in CRC, and its overexpression was closely associated with poor survival in patients with CRC. Consistent with our observations in CRC, a previous study in non-small cell lung cancer (NSCLC) also demonstrated that SLC25A1 expression is higher in primary and metastatic tumor tissues than in matched normal adjacent tissues [10]. Similarly, it has also been shown that the mRNA levels of CIC are increased in breast cancer cells [14]. These observations collectively suggest that SLC25A1 may play a critical role in the aggressiveness of various human cancers.

Marked upregulation of SLC25A1 in CRC suggests that SLC25A1 may function as an oncogene in the aggressiveness of CRC. Accordingly, the potential oncogenic function of SLC25A1 was investigated in both in vitro CRC cell lines and in vivo nude mouse models. Our results indicated that knockdown of SLC25A1 expression significantly inhibited proliferation and colony formation in LS174T and HT29X cells. In contrast, overexpression of SLC25A1 promoted cell proliferation and colony formation in SW620 and LOVO cells. In addition, injection of SLC25A1 knockdown LS174T cells into BALB/c mice further supported the role of SLC25A1 in the promotion of tumor growth in vivo. In agreement with our observations in CRC, it was also reported that knockdown of SLC25A1 in breast cancer by shRNA transfection remarkably blunted cell proliferation and colony formation in MBA-MD-231 cells [14]. Additionally, SLC25A1 also dramatically accelerated the in vivo growth of h1299 cells (lung cancer cell line) in xenograft models, while inhibition of SLC25A1 reduced the growth of lung cancer [14]. Moreover, another study in lung cancer also reported that inhibition of SLC25A1 could increase the sensitivity of ionizing radiation (IR) in NCI-H460 cells [15]. Consistently, we found that knockdown of SLC25A1 significantly inhibited CRC cell growth by suppressing the progression of the G1/S cell cycle and inducing cell apoptosis both in vitro and in vivo, whereas SLC25A1 overexpression suppressed these malignant phenotypes. These findings collectively indicate that SLC25A1 plays an important role in the promotion of tumor growth, and could serve as a therapeutic target for suppression of tumor growth.

We also investigated the role of SLC25A in the regulation of CRC metastasis. Both the migration and invasion of CRC cells did not change following knockdown or overexpression of SLC25A1 in CRC cells. Unlike our data in CRC that SLC25A1 has no effect on the metastasis of CRC cells, it was reported that inhibition of SLC25A1 significantly suppressed proliferation, migration and invasion of papillary thyroid carcinoma cells [16]. Inconsistencies may arise from different cancer types and cell line models, suggesting that the biological functions of SLC25A1may be cell type-specific.

Fatty acid metabolism reprogramming has been recognized as one of the hallmarks of malignancy, which serves as a key substrate for the generation of membranes, signaling molecules, and energy sources for rapid tumor growth [17, 18]. In recent years, studies have mainly focused on the roles of oncogenic genes and signaling in the promotion of abnormal lipid metabolism in human cancers [5]. However, the functional roles of SLC25A1, the only known transporter of human citrate, in the regulation of lipid metabolism remains unexplored in human cancer cells, including CRC. Here, we found that SLC25A1 significantly enhanced de novo lipid synthesis under normal conditions (nutrient-sufficient), which promoted CRC growth. Similar to our findings in CRC, a previous study in inflammatory steatohepatitis (NASH) also showed that suppression of SLC25A1 with CTPI-2 (a specific inhibitor of SLC25A1) reverted steatosis, glucose intolerance, and inflammation in the liver tissue [13]. These results suggest that SLC25A1 serves as a critical regulator of lipid metabolism in development and progression of NASH and cancers.

As solid tumors frequently outgrow their blood supply, the glucose concentration in tumor tissues is always lower than that in normal tissues [19, 20]. Therefore, it is critical for cancer cells to rewire their metabolic pathways to survive in nutrient-poor environments. Given that the functions of SLC25A1 include both the promotion of export of citrate from the mitochondria into the cytoplasm for fatty acid synthesis and the reverse import of cytosolic citrate into the mitochondria for OXPHOS, we hypothesized that SLC25A1 may play a role in the regulation of mitochondrial respiration in CRC during energy stress. An unexpected finding was that SLC25A1 had no significant effect on the oxygen consumption rate (OCR) and activity of mitochondrial respiratory chain complexes (I–V) under normal conditions (nutrient-sufficient). Nevertheless, SLC25A1 markedly elevated the OCR and activity of mitochondrial respiratory chain complexes (I–V) during energy stress. In addition, we further demonstrated that overexpression of SLC25A1 significantly attenuated starvation-induced cell apoptosis, while knockdown of SLC25A1 remarkably aggravated starvation-induced cell apoptosis. Given that AMP-dependent kinase (AMPK) is a critical energy stress sensor that reprograms energy metabolism by phosphorylating several transcriptional regulators, such as SREBP1, FOXO3, CHREBP1, HNF4α [21], the potential upstream genes modulating SLC25A1 may among these downstream transcriptional regulators of AMPK. However, considering that gene expression occurs at multiple levels, several other posttranscriptional and posttranslational regulators may also modulate SLC25A1 expression in CRC. Therefore, further research is needed to identify novel upstream regulators of SLC25A1 expression in CRC cells.

**Fig. 7:**
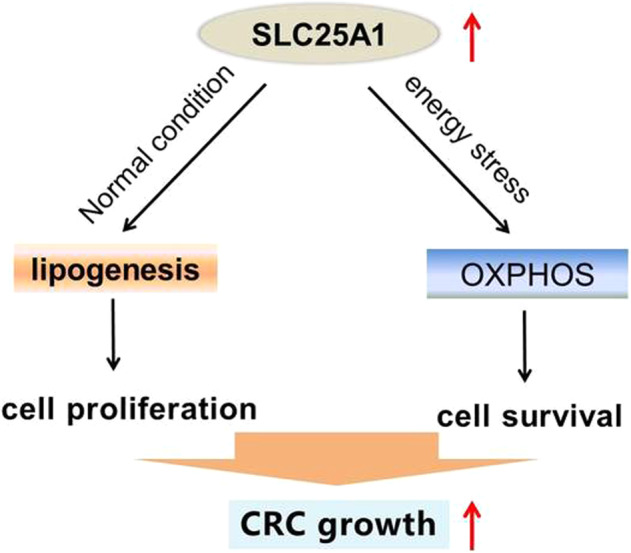
Schematic depicting the role of SLC25A1 in the metabolism reprogramming and progression of CRC.

Together, these findings suggest that SLC25A1 plays an important role in the dynamic metabolic switch between fatty acid synthesis and OXPHOS to promote tumor growth and maintain energy balance during energy stress (Fig. 7), implying that SLC25A1 could be a suitable target for metabolism-based therapeutic strategies in CRC.

## Supplementary information

Supplementary figures and table

The agreement from all authors to add Xianli He as a co-corresponding author

cddis_author_contribution_form

aj-checklist

## Data Availability

The authors declare that all data supporting the findings of this study are available from the corresponding author upon reasonable request.
