# Supplementary material for: SLC25A1 promotes tumor growth and survival by reprogramming energy metabolism in colorectal cancer
Source: Cell Death Dis. 2021 Nov 27;12(12):1108. doi: 10.1038/s41419-021-04411-2 (PMC8627508; doi:10.1038/s41419-021-04411-2)
Supplement: Supplementary file 1 — Supplementary figures and table [file 41419_2021_4411_MOESM1_ESM.docx]

**Supplemental information**

**SLC25A1 promotes tumor growth and survival by reprogramming energy metabolism in colorectal cancer**

**Supplemental figures**

**Figure S1. (A and B)** Cell migration and invasion abilities were determined in LS174T and HT29 cells with *SLC25A1* expression knocked-down. **(C and D)** Stable knockdown of *SLC25A1* was confirmed by qRT-PCR (C) and western blot (D) analysis in LS174T and HT29 cells. **(E and F)** Cell proliferation was determined by MTS (E) and colony formation (F) assays in SW620 and LOVO cells with *SLC25A1* expression stablely knocked-down.

**
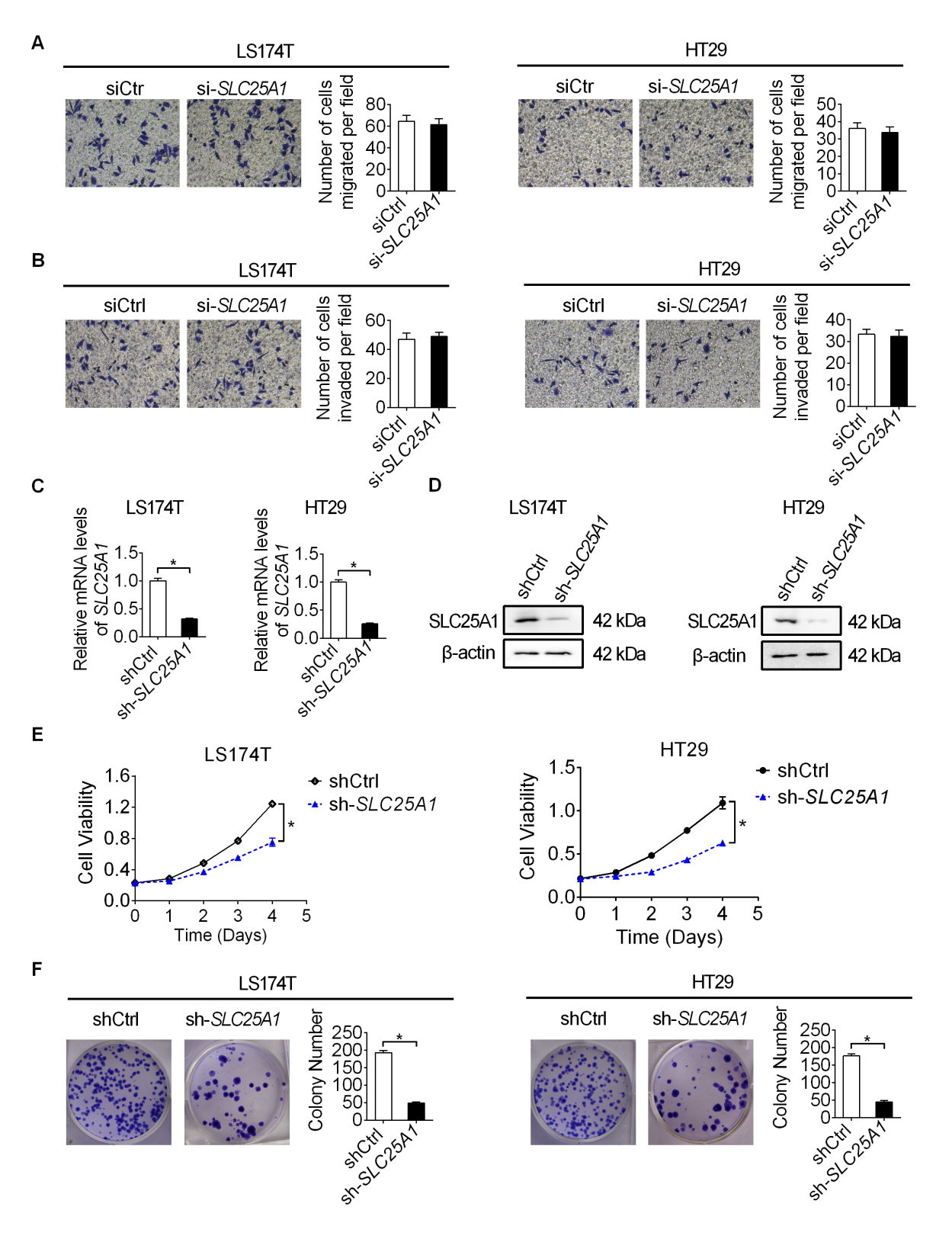
**

**Figure S2.** Fatty acid (FA) uptake was evaluated with a Free Fatty Acid Uptake Assay Kit in CRC cells with *SLC25A1* expression knocked-down or overexpressed.

**
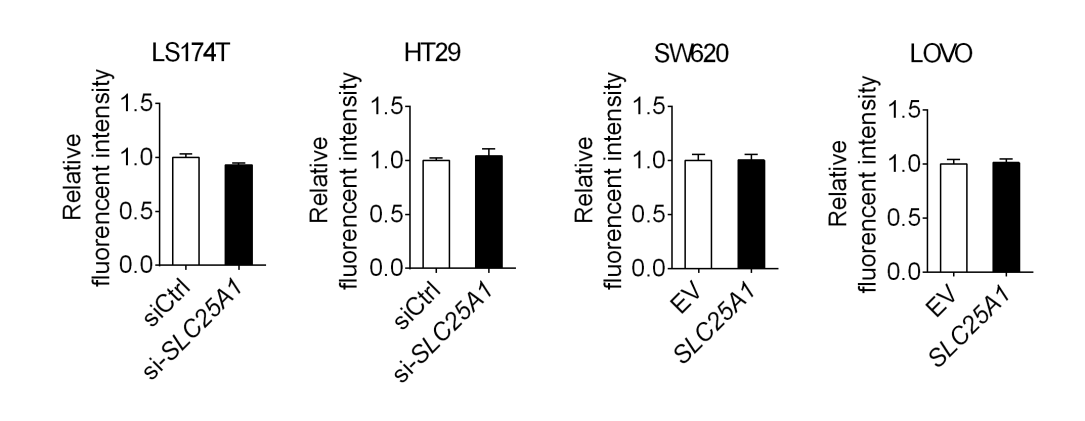
**

**Figure S3. (A)** Oxygen consumption rate (OCR) was determined in CRC cells under normal condition (in the presence of glucose). **(B)** Activities of respiratory chain complexes I-V were determined in CRC cells under normal condition (in the presence of glucose). **(C)** Mitochondria morphology was visualized by MitoTracker Green staining assay in CRC cells with *SLC25A1* expression knocked-down or overexpressed under normal or energy stress conditions.

**
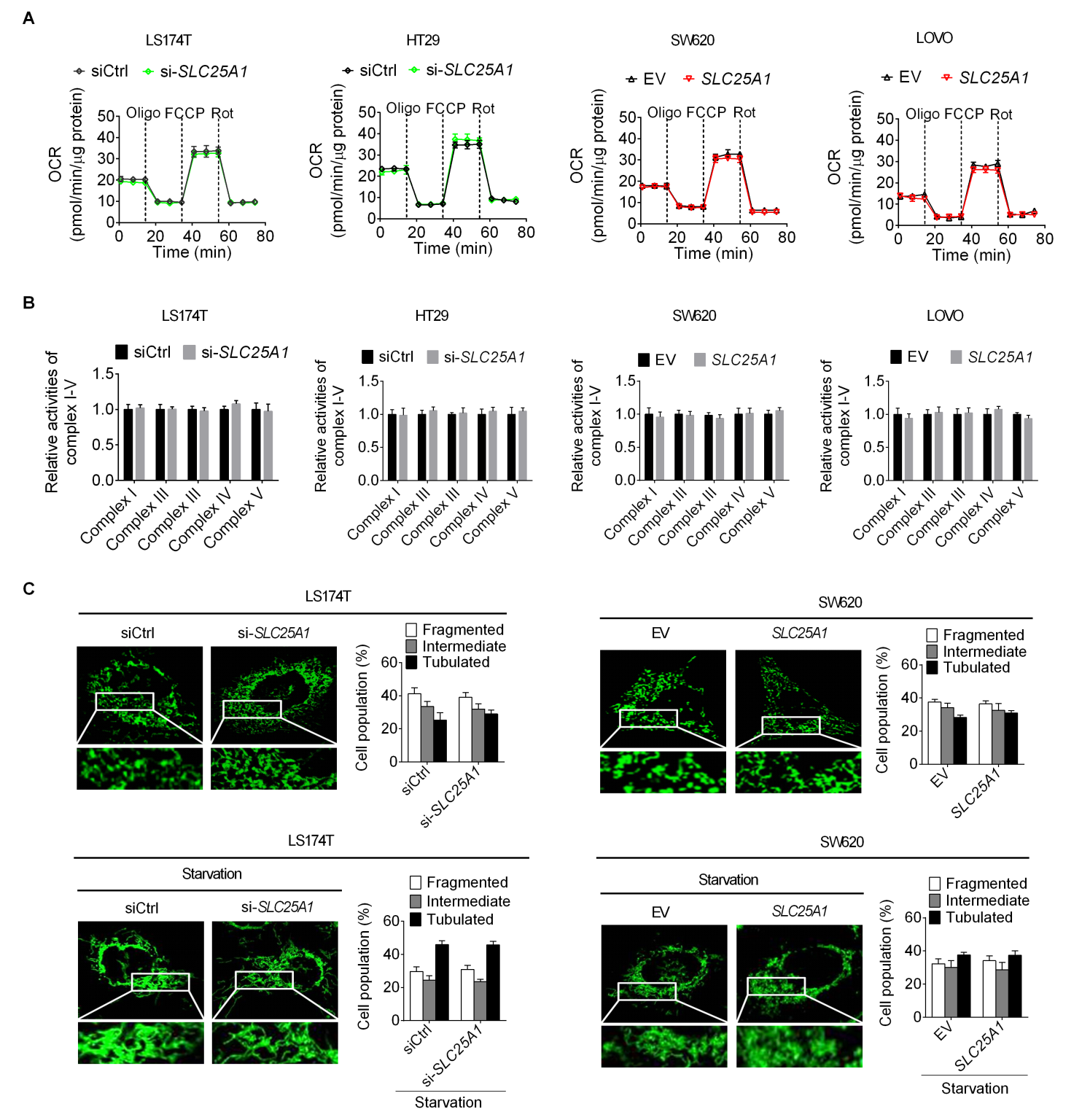
**

**Supplementary table 1. The relationship between SLC25A1 expression and clinicopathologic features of CRC patients**

| Variables | No. of cases (%) | SLC25A1 expression | | *P* value |
| --- | --- | --- | --- | --- |
|  |  | Low | High |  |
| All | 268 (100%) | 134 | 134 |  |
| Age |  |  |  |  |
| <60 | 147 (54.9%) | 75 | 72 | 0.806 |
| >=60 | 121 (45.1%) | 59 | 62 |  |
| Gender |  |  |  |  |
| Female | 155 (57.8%) | 74 | 81 | 0.458 |
| Male | 113 (42.2%) | 60 | 53 |  |
| Size |  |  |  |  |
| <5 cm | 142 (53.0%) | 83 | 59 | 0.005 |
| >=5 cm | 126 (47.0%) | 51 | 75 |  |
| Lymph node metastasis |  |  |  |  |
| No | 138 (51.5%) | 76 | 62 | 0.112 |
| Yes | 130 (48.5%) | 58 | 72 |  |
| Distant metastasis |  |  |  |  |
| No | 229 (85.4%) | 117 | 112 | 0.489 |
| Yes | 39 (14.6%) | 17 | 22 |  |
| Clinical stage |  |  |  |  |
| I+ II | 92 (34.3%) | 49 | 43 | 0.520 |
| III+ IV | 176 (65.7%) | 85 | 91 |  |
| Differentiation |  |  |  |  |
| Well+Moderate | 112 (41.8%) | 63 | 49 | 0.107 |
| Poor and others | 156 (58.2%) | 71 | 85 |  |
| Clinical stage |  |  |  |  |
| I+ II | 192 (71.6%) | 103 | 89 | 0.078 |
| III+IV | 76 (28.4%) | 31 | 45 |  |
| CEA |  |  |  |  |
| <5 ng/ml | 175 (65.3%) | 93 | 82 | 0.199 |
| >=5 ng/ml | 93 (34.7%) | 41 | 52 |  |
