# Supplementary material for: SLC25A1 promotes tumor growth and survival by reprogramming energy metabolism in colorectal cancer
Source: Cell Death Dis. 2021 Nov 27;12(12):1108. doi: 10.1038/s41419-021-04411-2 (PMC8627508; doi:10.1038/s41419-021-04411-2)
Supplement: Supplementary file 2 — The agreement from all authors to add Xianli He as a co-corresponding author [file 41419_2021_4411_MOESM2_ESM.pdf]

Re: Request agreement for addition of Xianli He as a co-corresponding author

发件人： Ying YANG<yingyang_dr@126.com>

收件人： wangnanafmu<wangnanafmu@163.com>

时　间：2021年11月06日 12:39 (星期六)

翻译成中文

你可以用泛微OA来审批... 点击启用

Prof. Nan Wang,

I agree to add Xianli He as a co-corresponding author.

Ying Yang
yingyang_dr@126.com

Ying Yang agree to add Xianli He as a co-corresponding author

Re: Request agreement for addition of Xianli He as a co-corresponding author

发件人： 贺加星<hejiaxing318@qq.com>

收件人： 王楠通讯<wangnanafmu@163.com>

时　间：2021年11月06日 13:19 (星期六)

翻译成中文

注册免费送云服务器 立即注册

I agree to add Xianli He as a co-corresponding author.

Jiaxing He agree to add Xianli He as a co-corresponding author

Re:Request agreement for addition of Xianli He as a co-corresponding author

发件人： doctorzb0207<doctorzb0207@163.com>

收件人： 王楠通讯<wangnanafmu@163.com>

时　间：2021年11月06日 12:39 (星期六)

翻译成中文

你可以用泛微OA来审批... 点击启用

I agree to add Xianli He as a co-corresponding author.

Bo Zhang agree to add Xianli He as a co-corresponding author

Re:Request agreement for addition of Xianli He as a co-corresponding author

发件人： 张战胜<zwsl123@163.com>

收件人： 王楠通讯<wangnanafmu@163.com>

时　间：2021年11月06日 13:54 (星期六)

翻译成中文

【企业出行】选曹操出行，省钱省力还省时！ 立即咨询

I agree to add Xianli He as a co-corresponding author.

Zhansheng Zhang agree to add Xianli He as a co-corresponding author

Re: Request agreement for addition of Xianli He as a co-corresponding author

发件人: 突然的自我<467421902@qq.com>

收件人: 王楠通讯<wangnanafmu@163.com>

时　间: 2021年11月06日 14:38 (星期六)

企业出行新客首充，充一返一，最高返100% 免费咨询

I agree to add Xianli He as a co-corresponding author.

Guozhan Jia agree to add Xianli He as a co-corresponding author

Re: Request agreement for addition of Xianli He as a co-corresponding author

发件人: 刘士祺<2843856726@qq.com>

收件人: 王楠通讯<wangnanafmu@163.com>

时　间: 2021年11月06日 12:48 (星期六)

翻译成中文

充10000返10000！曹操企业出行优惠福利多！ 免费咨询

I agree to add Xianli He as a co-corresponding author.

Shiqi Liu agree to add Xianli He as a co-corresponding author

回复: Request agreement for addition of Xianli He as a co-corresponding author

发件人: 鸡翅<504435076@qq.com>

收件人: 王楠通讯<wangnanafmu@163.com>

时　间: 2021年11月06日 13:03 (星期六)

给老板省钱！曹操企业出行，虚假报销0发生 免费咨询

Prof. Nan Wang

I agree to add Xianli He as a co-corresponding author.

Tao Wu agree to add Xianli He as a co-corresponding author

Re: Request agreement for addition of Xianli He as a co-corresponding author

发件人: wanghe<wanghe@fmmu.edu.cn>

收件人: 王楠通讯<wangnanafmu@163.com>

抄送人: yingyang_dr<yingyang_dr@126.com> 176228342<176228342@qq.com> doctorzb0207<doctorzb0207@163.co

存所有抄送人

时　间: 2021年11月08日 09:26 (星期一)

翻译成中文

你可以用泛微OA来审批... 点击启用

I agree to add Xianli He as a co-corresponding author.

Xianli He agree to add himself as a co-corresponding author
