# Supplementary material for: SLC25A1 promotes tumor growth and survival by reprogramming energy metabolism in colorectal cancer
Source: Cell Death Dis. 2021 Nov 27;12(12):1108. doi: 10.1038/s41419-021-04411-2 (PMC8627508; doi:10.1038/s41419-021-04411-2)
Supplement: Supplementary file 3 — cddis_author_contribution_form [file 41419_2021_4411_MOESM3_ESM.pdf]

DECLARATION OF CONTRIBUTIONS TO ARTICLE **ADMC**

Manuscript Number:	Journal Name:	
CDDIS-21-3271RR	*Cell Death & Disease*	(the 'Journal')

Proposed Title of the Contribution:

SLC25A1 promotes tumor growth and survival by reprogramming energy metabolism in colorectal cancer (the 'Contribution)

Author(s):

Ying Yang, Jiaxing He, Bo Zhang, Zhansheng Zhang, Guozhan Jia, Shiqi Liu, Tao Wu, Xianli He, Nan Wang (the 'Authors')

For all *CDDis* articles, each person named as an author in the published version must be able to show he or she has contributed substantially to the article.

Authorship credit should be based on 1) substantial contributions to conception and design, acquisition of data, or analysis and interpretation of data; 2) drafting the article or revising it critically for important intellectual content; and 3) final approval of the version to be published. Authors should meet conditions 1, 2 and 3.

Any person who cannot be shown to have made a substantial contribution to the article cannot be listed as an author in the final version. The name of any person who is deemed to have made a minor contribution can, however, appear in the Acknowledgments section of the article.

Please complete the table below to indicate the contributions of all named authors to the manuscript.

Author Full Name:	Specification of Contribution to the Manuscript:
Ying Yang	Wrote the paper, performed most of the experiments and analyzed the data
Jiaxing He	Performed most of the experiments and analyzed the data
Bo Zhang	Performed most of the in vitro experiments and analyzed the data
Zhansheng Zhang	Performed most of the in vitro experiments and analyzed the data
Guozhan Jia	Performed most of the in vivo experiments and analyzed the data
Shiqi Liu	Performed most of the in vivo experiments and analyzed the data
Tao Wu	Designed the overall study and supervised the experiments
Xianli He	Designed the overall study and supervised the experiments
Nan Wang	Designed the overall study and supervised the experiments. revised the paper and acquired the funding

Please complete the table below to indicate the contributions of all named authors to the figures.

Figure 1:

G. J and S. L generated the data, J. H performed the statistical analysis, Y. Y assembled the figure

Figure 2:

G. J and S. L generated the data, J. H performed the statistical analysis, Y. Y assembled the figure

Figure 3:

Y. Y, J. H, B. Z. and Z. Z generated the data, J. H performed the statistical analysis, Y. Y assembled the figure

Figure 4:

B. Z. and Z. Z generated the data, J. H performed the statistical analysis, Y. Y assembled the figure

Figure 5:

Y. Y, J. H, B. Z. and Z. Z generated the data, J. H performed the statistical analysis, Y. Y assembled the figure

Figure 6:

Y. Y, J. H, B. Z. and Z. Z generated the data, J. H performed the statistical analysis, Y. Y assembled the figure

Y. Z generated the graphic abstract

Signed for and on behalf of the Author(s):	Print Name:	Date:
Nan Wang	Nan Wang	Nov 4th, 2021
